# Patient experiences and expectations of faecal immunochemical testing for investigation of colorectal cancer symptoms: a cross-sectional qualitative interview study with patients and practitioners in the UK

**DOI:** 10.1136/bmjopen-2024-093215

**Published:** 2025-05-16

**Authors:** Adam Biran, Christina Dobson, Colin J Rees, Willie Hamilton, David Humes, Laura Jane Neilson, James Turvill, Christian von Wagner, John Whelpton, Linda Sharp

**Affiliations:** 1 Population Health Sciences Institute, Newcastle University, Newcastle upon Tyne, UK; 2 British Society of Gastroenterology, London, UK; 3 University of Exeter Medical School, Exeter, UK; 4 Gastrointestinal and Liver Theme, NIHR Nottingham Biomedical Research Centre, Nottingham, UK; 5 Nottingham University Hospitals NHS Trust, Nottingham, UK; 6 Queen’s Medical Centre, University of Nottingham School of Medicine, Nottingham, UK; 7 South Tyneside and Sunderland NHS Foundation Trust, South Shields, UK; 8 York and Scarborough Teaching Hospitals NHS Foundation Trust, York, UK; 9 Department of Epidemiology and Public Health, University College London, London, UK; 10 Lay Investigator, Newcastle University, Newcastle upon Tyne, UK

**Keywords:** QUALITATIVE RESEARCH, Primary Health Care, Adult gastroenterology, Endoscopy, Adult oncology

## Abstract

**Abstract:**

**Objectives:**

Faecal immunochemical testing (FIT) is now commonplace in the UK to prioritise symptomatic patients for urgent gastrointestinal investigation. The test requires a stool sample to be collected at home by the patient and returned for analysis. In this qualitative study, we sought to understand the feasibility and acceptability of FIT-based triage for patients.

**Design:**

A cross-sectional, qualitative, experiential interview study.

**Setting:**

Recruitment was through three participating UK NHS sites (Yorkshire, Midlands, North-East). Health professionals were also identified through membership of the BSG/ACPGBI Symptomatic FIT Guideline Development Group and snowball sampling.

**Participants:**

We interviewed 21 patients who had completed FIT and been referred for colonoscopy and 30 primary and secondary care health professionals involved in symptomatic FIT delivery.

**Results:**

Completion of FIT was unproblematic from the perspective of patients who returned the test. However, health professionals expressed concern over non-return. Among patients, understanding of the purpose of FIT and the meaning of results varied. Health professionals acknowledged that ensuring patient understanding of these can be challenging. Patients believed colonoscopy was less likely to miss cancer than FIT. Patients with a family or personal history of cancer were particularly anxious and wanted the reassurance of colonoscopy, even with a negative FIT result.

**Conclusions:**

We found no major barriers to the use of FIT in prioritising symptomatic patients for urgent investigation. Improving communication might increase compliance and, possibly, acceptability of non-referral for colonoscopy in the case of a negative test result.

**Strengths and limitations of this study**

- Our sample included patients with positive and negative faecal immunochemical testing (FIT) results drawn from several regions.
- The inclusion of data from interviews with health professionals involved in symptomatic FIT delivery provided a complementary perspective on communication around FIT and FIT results and provided insights (although indirectly) into the experiences of a wider patient group, including those not referred for further investigation after FIT.
- Eligibility of patients was restricted to those who had completed a FIT and received colonoscopy; further study is merited of symptomatic patients who declined to complete a FIT or who were not referred for urgent investigation, whose experiences may be different.
- Only one participant self-identified as non-White; it is possible the experiences of symptomatic FIT among patients from minority ethnic groups may differ from those of White patients.
- Our sample of health professionals included members of the BSG/ACPGBI Symptomatic FIT Guideline Development Group whose views and experiences may not be representative of the wider population of health professionals.

## Introduction

 Each year in the UK, more than 800 000 colonoscopies are conducted.^1^ One major indication for colonoscopy is investigation of symptoms of possible colorectal cancer (CRC). Currently, fewer than 2% of procedures in symptomatic individuals in the UK result in a CRC diagnosis.^2^ For patients, undergoing colonoscopy can provoke considerable anxiety,^3^ and there is a small risk of adverse effects, including perforation and significant bleeding.^4^ Colonoscopy demand in the UK has been rising year-on-year, with endoscopy services failing waiting time targets. This has focused interest on finding ways to identify those patients at highest risk of CRC, who could most benefit from urgent colonoscopy. Increasing colonoscopy appropriateness could increase the proportion of patients with relevant findings, reducing unnecessary examinations and costs.^5^

Faecal immunochemical testing (FIT) is used for population-based CRC screening in the UK. During the COVID-19 pandemic, FIT was increasingly used in the symptomatic population to help define and prioritise patients for access to colonoscopy. In 2022, guidelines were published for use of FIT in patients presenting in primary care with features of possible CRC to determine which patients require further investigation.^6^

FIT measures haemoglobin (Hb) concentrations within a stool sample. This sample is collected by the patient, using a dedicated kit. For FIT-based referral in England, kits are usually sent to symptomatic patients following a request from their general practitioner (GP). The completed sample is then posted by the patient to a laboratory where the faecal Hb concentration is measured and compared against a nationally defined cut-off. Currently, ≥10 µg Hb/g faeces is considered ‘positive’. Patients with a positive FIT result are referred to secondary care for urgent investigation, most usually by colonoscopy or computed tomographic colonography. Patients with a negative FIT result may still be referred into secondary care but usually on a routine (ie, non-urgent) pathway.

Sustained, successful implementation of FIT-based referral of patients with symptoms of possible CRC requires acceptance and active participation from patients. In asymptomatic population-based screening populations, barriers to FIT completion, and screening participation more generally, have been reported (see, for example^7^,^9^). To date, few studies (qualitative or quantitative) have investigated FIT among symptomatic patients, whose understanding, preferences, health behaviour motivations, and experiences may differ.^10^,^13^

Given the relative paucity of the evidence base, the objectives of this qualitative study were to explore, in the context of symptomatic FIT: patient understanding of the test; experiences of test completion; understanding and communication of test results; and expectations with respect to investigation in secondary care.

## Methods

### Study design

This was a cross-sectional, qualitative, experiential, interview study undertaken within the UK.

### Eligibility and recruitment

Patients were eligible if they had had a colonoscopy between 3 and 12 months previously, following referral from primary care after presentation with bowel symptoms and completion of a FIT (regardless of the results). Eligible patients were identified from historical colonoscopy lists by health professionals at three participating UK NHS Trust sites chosen for socio-economically and/or ethnically diverse catchment populations (Yorkshire, the Midlands and the North-East). Participants were required to be sufficiently proficient in the English language to take part in an interview, without an interpreter.

Eligible patients were provided with a study information sheet and consent form (both documents were in English). Those interested returned a completed form to the research team, who contacted them to arrange an interview. Patients who completed an interview were offered a £25 shopping voucher to thank them for their participation.

Primary and secondary care health professionals were recruited primarily to explore FIT implementation issues reported elsewhere.^14^ However, data from this participant group were integrated into this analysis to provide complementary perspectives on communication around FIT testing and test results, and practitioner reflections on patient acceptability and challenges. Health professionals were eligible if they were involved in delivery of symptomatic FIT pathways, including primary care, secondary care and pathology services. Eligible professionals were identified by the three participating sites as well as through membership of the BSG/ACPGBI Symptomatic FIT Guideline Development Group^6^ and snowball sampling. Eligible professionals were sent an invitation letter and information sheet and expressed their willingness to take part by emailing the research team. A team member then contacted willing individuals to arrange an interview. Informed consent was given verbally, immediately prior to interview and was audio-recorded.

### Data collection

Data were collected through semistructured interviews with patients and health professionals. Interviews were carried out remotely by phone or online (using Microsoft Teams) and lasted between 20 and 60 min. Interviews were conducted following a topic guide (different for each participant group) developed by the research team and lay representatives (see online supplemental file 1). Topics explored with patients included history of symptoms, reason for attending primary care, understanding of FIT and of FIT results, experience of completing FIT and expectations regarding referral to secondary care. For health professionals, topics included patients’ understanding of FIT and of FIT results, patients’ ability to complete FIT and patients’ expectations regarding referral to secondary care (other topics relating to health professionals’ experiences of implementing FIT and their perceptions of the benefits and consequences of symptomatic FIT were also explored and are reported in a separate paper^14^). Topic guides were applied flexibly to allow changes in question order and exploration of new topics arising during the interviews. Health professional interviews were conducted by one of two researchers (CD (female) and AB (male)). CD and AB are experienced, qualitative, health researchers with expertise in colorectal cancer pathways and have non-clinical backgrounds. Patient interviews were conducted by AB only. There is a possibility that this might have had a differential effect on the data provided by male and female respondents. Interviews were conducted between September 2022 and September 2023. Data collection ceased when the researchers (AB and CD) believed that additional interviews were no longer generating new data that would contribute additional insights.

### Data handling and analysis

Interviews were audio-recorded, transcribed verbatim, pseudonymised and imported into NVivo for coding. Data were analysed using a framework approach.^15^ The framework was initially derived from the topic guide by AB and reviewed for usefulness and comprehensiveness by CD and LS. Three transcripts from each participant group were coded independently by CD and AB and discussed to check the appropriateness of the coding structure and ensure broad agreement on interpretation of the data. Additional codes were added during analysis and applied retrospectively to transcripts already coded. Coding of the patient interviews was carried out by AB and reviewed by CD and LS. Healthcare professional interviews were coded by AB and CD and discussed and reviewed with LS.

### Data availability statement

The data that support the findings of this study are available from the corresponding author on reasonable request as a pseudonymised dataset.

### Patient and public involvement

A patient panel of individuals with experience of FIT and endoscopy contributed to study oversight from conception to dissemination; best practice principles, as set out in the UK Standards for Public Involvement, were followed throughout. Specifically, panel members reviewed patient-facing study documents (information sheet, consent form), and draft interview guides, contributed to interpretation of data and theme development, co-produced lay summaries and commented on an earlier draft of the manuscript.

## Results

We interviewed 21 patients (12 women, 9 men) who had experienced symptoms of possible CRC, completed a FIT in primary care and undergone colonoscopy. They had a median age of 67 years (range 28–86); 20 identified as White. We interviewed 30 health professionals from a variety of specialties (table 1). Ten of these participants had been members of the BSG/ACPCBI Symptomatic FIT Guideline Development Group.

**Table 1:** Characteristics of health professional sample

Specialty	Number of participants
GP	7
Gastroenterologist	7
Surgeon	6
Nurse endoscopist	2
Gastroenterology nurse co-ordinator	1
Consultant nurse	1
Nurse practitioner	1
Inflammatory bowel disease nurse	1
Colorectal cancer and stoma nurse	1
Pathology lab director	1
Consultant biochemist	1
Radiologist	1

GP, General Practitioner; GP, general practitioner.

We organised our findings around four themes reflecting the broad sequence of steps in the process of FIT-based referral: Understanding the Test; Practicalities of FIT Completion; Understanding the Test Result; and Expectations of Referral and Investigation. Themes are outlined below, and illustrative quotes presented. Additional data can be found in online supplemental table 1.

The first theme was ‘Understanding the Test’. This brought together perspectives on what patients believed the test assessed, its purpose and how this was communicated by professionals.

I don’t really know…they can detect cancer through it, I think. (P003, male, mid-70s)

Some patients do come to us thinking that it’s a test for cancer, and we have to tell them that it’s not… Some patients become very anxious. (C016, Colorectal Consultant Nurse)

There was variation in the way in which FIT was understood by patients, with some believing it to be a test for cancer, while others understood it was a test for the presence of blood. This was reflected in interviews with health professionals. Some health professionals reported that their available time or ability to explain FIT to patients was limited. One GP explained that they sometimes presented FIT to patients as a cancer screening test, believing this would increase the likelihood of completion.

I do say it’s a cancer screen because I think it makes it more likely for the patient to do it. (CO17, GP)

Other professionals reported that some patients did not differentiate between FIT for population-based screening and FIT used as a symptomatic triage tool: consequently, patients who had recently completed a screening FIT could be reluctant to complete what they regarded as an unnecessary repeat test.

The second theme ‘Practicalities of Test Completion’ explored the process of collecting the stool sample.

The tube wasn’t great with the little stick. I think the tube itself needs to be a little bit thicker; but other than that, the process of doing it was fine. (P012, female, mid-40s)

… our instructions are very pictorial but there’s still that language barrier, even if English is your first language…People with learning disabilities might not understand as well. (C028, Pathology Lab Director)

Successful FIT completion requires the patient to read and understand the instructions for taking, labelling and returning the sample, as well as having the ability to physically complete the sampling procedure. Some concerns were voiced by patients and health professionals in relation to each of these requirements. While all the participating patients found the FIT instructions easy to follow, professionals working in pathology laboratories reported queries from patients about completion; they described receiving incorrectly completed samples, suggesting poor comprehension of instructions by some patients. Health professionals had some concerns about the ability of patients with learning disabilities, or for whom English was not their first language, to understand the instructions. Health professionals also voiced concerns about the ability of patients with visual or fine motor impairments to complete FIT.

There’s the visually impaired, I think they really struggle to do the test. (CO28, Pathology Lab Director)

These concerns were echoed by some patients who referred to the small diameter of the FIT sample tube as a potential barrier to successful FIT completion.

Notwithstanding the suggested challenges of sample collection for patients with impaired manual dexterity, participating patients found the test easy to complete (although “not the pleasantest of things”, P002, male, 76 years). Health professionals also reported that most patients they encountered in clinical practice were happy with the process of completing FIT. Nevertheless, non-completion, or non-return of samples, was a concern raised by several health professionals, with one laboratory director estimating a non-return rate of around 20%.

The third theme ‘Understanding the Test Result’ explored the ways patients interpreted the FIT result and how it was communicated to them by professionals.

you presume obviously there’s a problem there of some sort. You just hope it’s not as serious as it might be. (P002, male, mid-70s)

Everything was negative, there was no cancer they’d detected. That’s what I presumed it was… I don’t have bowel cancer, I’ve been tested for it, well, I’m presuming that’s what it was doing and so I don’t have bowel cancer or anything, so then obviously they have to look at somewhere else to find the reason why I’ve lost so much iron. (P010, female, mid-50s)

I’ve never really given them the score (Hb concentration) because I don’t think it means very much, well, because it doesn’t really mean very much to me either! … I would just phone them up and say, “we’ve had your poo test back, it’s positive, therefore we’re going to go into the next stage”, so I think you’re best to have somebody actually look at your bowel. (C009, GP)

There was variation in patients’ understanding of results. A negative result was interpreted in various ways, including as meaning there was no blood in the sample, or that there was no cancer present; other patients expressed uncertainty. Patients generally understood a positive result as indicating a potential problem which could be cancer, as opposed to a definitive finding of cancer. However, health professionals reported examples of patients who had believed a positive FIT result meant that they had tested positive for CRC. Health professionals (primarily GPs) communicated FIT to patients as positive or negative results rather than as concentrations. Some health professionals sought to reassure patients about the low probability of them having cancer in the context of a negative FIT result.

The fourth theme was ‘Expectations of Referral and Investigation’. This theme considered patients’ wishes in relation to and satisfaction with the investigation of symptoms.

I don’t think I’d have settled without it [a colonoscopy]. Because I knew from my symptoms there was something going on, there was something not right. I had no idea what it was. Polyps and things just hadn’t crossed my mind…just cancer (P012, female, mid-40s)

you do sometimes find that you’re perhaps saying to people, actually no you don’t need to be investigated where they desperately want to get through. And then other people, actually I need to send you through, and they just don’t want to go anywhere near secondary care. (C020, GP)

Patients reasoned that because colonoscopy is a visual inspection, it must be more thorough than FIT and less likely to miss cancer. One health professional suggested that this perception was reinforced in the explanations provided in patient literature about the investigation of gastrointestinal symptoms which might not make patients aware that colonoscopy can also miss cancers.

For some patients with a family, or personal, history of cancer, the reassurance of a colonoscopy was something they sought from their presentation in primary care. These patients believed that they would have been dissatisfied had they had a negative FIT and not been referred. However, other patients with a positive FIT reported that they were content to rely on the judgement of a health professional and believed that they would have been content not to be referred for colonoscopy had their FIT been negative.

Health professionals reported individual variation in patient preferences with respect to gastrointestinal investigations. Some patients were sufficiently reassured if told their FIT result suggested no further investigation for cancer was warranted; professionals also noted that these patients might be relieved to avoid an invasive investigation. However, professionals also stated that other patients still wanted the reassurance they anticipated would come from having a colonoscopy.

Referral into secondary care was not only seen as a means of diagnosing, or excluding, cancer. Some patients also saw this as a route to specialist care, access to which would allow them to explain, and receive help in addressing, their bowel symptoms. Health professionals noted that patients want to know the cause of their symptoms, as well as what measures will control or eliminate them. Therefore, simply reassuring these patients that their symptoms were unlikely to be the result of an underlying cancer was not sufficient.

## Discussion

This qualitative study explored symptomatic FIT completion, including patient understanding of the test, experiences of test completion, understanding of test results, and expectations of referral and investigation. We drew on the perspectives of individual patients who had completed symptomatic FIT and health professionals with experience of caring for multiple patients through their involvement in symptomatic FIT pathways.

We found no evidence for substantial barriers to the use of FIT-based referral, either in relation to test completion or acceptability of it as a triage tool, within this participant group who had completed FIT. Consistent with previous studies of FIT in symptomatic populations,^10 11 13^ and asymptomatic screening populations,^8 9^ none of our participants reported significant difficulties with completing FIT sampling. However, some participants expressed doubts over the comprehensibility of instructions for particular patient groups and suggested it could be challenging for patients with visual or fine motor impairments to use the kit. Previous work^13^ has shown that these issues can often be overcome with sufficient planning and preparation; nevertheless, they might underlie some of the spoilt samples and test non-return reported as a concern by some health professionals in our study. Work in the context of asymptomatic screening suggests that, for some individuals, video may be useful to improve the clarity of FIT instructions.^12^ It would be valuable to evaluate this and other ways to provide/convey test instructions (eg, static or moving animations, photographs on leaflets, encouragement to use instruction helpline) in the context of symptomatic FIT. If barriers to correct completion and return of stool samples are addressed, this may improve patient experience, help avoid diagnostic delays and increase the likelihood of future FIT completion.^10^

Participating patients varied in their understanding of the purpose of FIT and of meaning of FIT results. Although some patients perceived FIT as being a test for cancer, participants with a positive FIT result did not report interpreting this as meaning they *definitely* had cancer. This may indicate that patient understanding of FIT is generally good. This interpretation is consistent with the findings of Delisle *et al*^10^ who surveyed more than 1100 people who had been referred for colonoscopy between December 2018 and July 2019 and who were invited to complete a symptomatic FIT as part of a research study; 98% of participants agreed that they understood what the test was being used for. However, it should be noted that, in that study and the current study, by the time of survey completion or interview, participants had already either been triaged for colonoscopy (Delisle *et al* study) or attended secondary care for colonoscopy (current study); in both instances, health professionals may have further explained the FIT test and its purpose, and the purpose of colonoscopy. Moreover, some GPs in the current study mentioned that some patients may misunderstand positive test results and interpret these as indicating the presence of cancer; similarly, some patients interviewed stated that they believed that a negative test result meant that they did *not* have cancer. These findings indicate that at least some of those who complete a symptomatic FIT lack a good understanding of what the results mean. To address this, consideration should be given to improving patient information around the test and its results.

Health professionals noted that it could be difficult to explain FIT purpose or results to patients in the context of short consultation times and heightened patient anxiety. Health professionals reported that patients did not always understand the difference between FIT for population-based screening and symptomatic FIT, and that a recent experience of screening FIT could result in patients being unwilling to complete a symptomatic FIT, regarding this as pointless repetition, despite symptomatic and screening FITs having vastly different thresholds for ‘positive’ results. Our findings suggest a lack of clarity when symptomatic FIT is explained to patients, which may influence test completion as well as patient understanding. Similar communication issues were identified in the qualitative study by Snudden *et al*,^11^ in which participants noted that explanations by professionals about what FIT was, and why it was indicated, tended to be poor. The participants in that study were interviewed between April and October 2020 when COVID-19 restrictions were at their height; it might have been assumed, therefore, that the communication issues reported in that study were a result of the disruptions and abrupt changes to ways of engaging with GPs during that period. That appears to be incorrect as our work has shown that these communication difficulties persist and are recognised by professionals. Improved communication around FIT, including highlighting the distinctions between the application of the test in screening and symptomatic contexts, might improve compliance and decrease patient anxiety.

Some health professionals in our study reported concerns about non-completion of FIT and one estimated a non-return rate of around 20%; if correct, this could have important consequences both for the health services and individual patients. Formal, quantitative, assessment of FIT completion rates was beyond the scope of this study. However, we suggest that routine monitoring of symptomatic FIT (non-)completion—including identification of whether particular subgroups of the population are more, or less, likely to complete a FIT—could generate valuable insight into how to improve symptomatic FIT delivery.

Participants had greater confidence in colonoscopy than FIT, and our data suggested there may be a group of patients, characterised by a personal or family experience of cancer, who want the reassurance of a colonoscopy, irrespective of FIT result. This finding contrasts with that of Gill *et al*^16^ who found that anxious patients with a personal or family history of CRC experienced reassurance from a negative FIT result, though the studies used different methods, and the results are not directly comparable as we did not measure anxiety. Moreover, our participants may also have experienced some reduction in anxiety on receipt of a negative FIT result but continued to want further reassurance, or explanation for their symptoms, from a colonoscopy. Banks *et al*^17^ found, among a sample of the public, a preference for diagnostic cancer testing at lower levels of risk than those proposed in clinical guidelines, an effect that was more pronounced in those with a family history of cancer. In our study, patients with cancer experience had often entered primary care wanting or expecting a referral for colonoscopy. Thus, while (as has been suggested elsewhere^18^) most patients may prefer FIT to the option of colonoscopy, the preferences of some subgroups may be different. Additional communication may be needed to reassure these groups if a referral for colonoscopy is not offered.

Furthermore, patients enter the health system with symptoms for which they seek not only explanation, but also, remedy. Even though a negative FIT result can be reassuring with respect to cancer, the data reported here suggest that an explanation for, or help with, symptoms is still needed. This point was also raised in our public and patient involvement work predating the study. The guidance for use of symptomatic FIT recommends that safety-netting mechanisms be put in place for patients with a negative FIT who have continuing or worsened symptoms, and this group should be considered for specialist referral, perhaps using the non-specific symptoms pathway.^19^

## Conclusions

We found no evidence to suggest substantial barriers to the use of FIT as a viable approach to support prioritisation of referrals and resources for colonoscopy. However, better communication of the purpose of FIT might help reduce anxiety in some patients. Consideration is needed as to how to best manage and support patients with a personal or family history of cancer if they are not referred for further investigation following a negative FIT result.

Effective communication around FIT is challenging within the constraints of the UK NHS, in which primary care consultation times are typically short.^20^ Future work might look more closely at the content and format of communication around FIT with a view to optimising the information provided by health professionals to reduce possible confusion (and, potentially, FIT non-completion).

Future work might also investigate the experiences of patients who were not referred into secondary care after a negative FIT result and those who were referred through an alternative pathway, such as that for non-specific symptoms, both in terms of management of their symptoms and satisfaction with their care.

## Supplementary material

10.1136/bmjopen-2024-093215 online supplemental file 1

10.1136/bmjopen-2024-093215 online supplemental table 1

## Data Availability

Data are available upon reasonable request.
