# Supplementary material for: Patient experiences and expectations of faecal immunochemical testing for investigation of colorectal cancer symptoms: a cross-sectional qualitative interview study with patients and practitioners in the UK
Source: BMJ Open. 2025 May 16;15(5):e093215. doi: 10.1136/bmjopen-2024-093215 (PMC12086939; doi:10.1136/bmjopen-2024-093215)
Supplement: online supplemental file 1 [file bmjopen-15-5-s001.docx]

**Indicative Interview Topic Guide: Patients**

*This guide covers the general topics and questions that will be covered in the interviews. It will be used flexibly, so that the participant can share information in a way that is most natural to them. Questions are intended to be used with appropriate probes as needed. How questions are asked and the order in which they will be asked will vary from interview to interview. It may also be the case that not all topics and questions are covered in every interview.  If a new topic of interest arises in one interview, this may be added to the guide for inclusion in subsequent interviews. Conversely, a topic or question that is found not to deliver useful information may be dropped from subsequent interviews.*

***Symptomatic Experience & Background***

- To begin with, please can you tell us a bit about the symptoms you have been experiencing/experienced?
  - What symptoms did you have?
  - How long had they been going on?
- What did you think might be causing your symptoms?
- What made you decide you needed to see your doctor about them?
- What did you hope for or want your GP to do for you?
  - Why / why was this important to you?
  - Did you expect that they would do this for you?
  - Why / why not / what did you expect would happen?

***Primary Care Experience***

- When you had your first appointment, was it a GP or nurse you spoke to/saw?
- What did they say might be the cause of your symptoms?
- What did your doctor, or nurse, do for you? For instance, did they make a referral to the hospital, give you a prescription, or ask you to come back if your symptoms didn’t improve?
- **How did you feel about this? / Why?**

Some people are asked to provide poo samples, or do a poo test, by their GP, and others are asked to provide them to the hospital, before they go for their investigations. There are different types of poo sample, We are interested in the kind where you are given a kit with a container for you to take home, collect a sample of your poo and send it back in a special envelope. The poo is tested looks for traces of blood in the poo. This can sometimes be a sign of bowel cancer.

- Did your GP ask you to provide a stool/poo sample like this?

If so, ask the following questions:

- - - How did you feel about giving the sample?
    - How easy or difficult was it to do?
    - Was there anything that could have helped to make it easier?
    - At the time, what did you understand this was for?
      - Is that still your understanding now?
      - How did you feel about this?
    - What did you expect would happen after giving this sample?
      - Is that what happened?
    - How long did you wait for your test result?
      - How did you find out your test result (i.e. a phone call, letter, or the patient rang up to find out)?
      - What did you think the test results meant? (do they interpret this in terms of certainty / probability / risk – e.g. they don’t have cancer v they probably don’t have cancer)
      - Did anyone explain to you what this test result meant?
      - Who was that? What did they tell you?
      - **(If Not had result -** How did you feel about this?
      - Would you have preferred to know the result?
    - Sometimes a GP uses this test to help decide who should be sent for colonoscopy and who should not. What do you think about this?
    - If your GP had let you choose, either to be referred for a colonoscopy, or to give a poo sample first and use that to decide whether to refer you for a colonoscopy, which would you choose? Why?
    - If your GP had suggested that it would be best not to refer you for colonoscopy because your poo sample, when it was tested in the lab, had a “low score”, how do you think you would have felt about this? Why?
    - [If anxious / unhappy] What do you think you would want the GP to do if your test score was low?

If not, ask the following questions:

If your doctor asks for you to provide a poo sample, you are given a kit with a container for you to take home, collect a sample of your poo and send it back in a special envelope. The test looks for traces of blood in the poo, which is a sign of bowel cancer (but not caused by cancer in all cases).

- - How do you think you would you have felt if your doctor had asked you to provide a poo sample, for them to do this test?

The GP might use the results of this test to help decide whether or not it would be best to refer to the hospital for further investigations. Generally, if the test result comes back as ‘high’, then they refer patients to the hospital for further investigations (usually a colonoscopy). If the result comes back as ‘low’ the GP wouldn’t always refer patients to the hospital.

- - Do you think that you would have been happy for the doctor to have used this test result to make decisions about your care?
  - If you had had a ‘'low’ score on this test. Would you have been content not to have investigations at the hospital?
    - Would you have had any concerns about this?

What would you have wanted the GP to do next?

- Do you remember how many times you went to your doctor about your symptoms before you were referred to hospital?

***Secondary Care Experience***

- Was this the first time you had been for a colonoscopy?
- Did you have an appointment to see a doctor at the hospital to discuss your condition before your procedure, or was your colonoscopy your first hospital appointment in relation to your symptoms?

Some people are asked to provide poo samples by their GP, and others are asked to provide them to the hospital, before they go for their investigations. While you were waiting for your colonoscopy/CTC appointment, were you asked to provide a poo sample?

- If so, ask them:
  - At the time, what did you understand the test was for?
  - Is that still your understanding now?
  - What did you expect would happen after taking the test?
  - Is that what happened?
  - How did you feel about taking the test?
  - How easy was it to do the test?
  - Was there anything that could have helped to make it easier?
  - How did you feel about the fact that the doctor was using this to help guide decisions about your care?
  - Were you told your test result?
  - **(If yes)** How did they tell you this (i.e. a phone call, letter, or the patient rang up to find out)?
  - What did you think the test results meant?
  - Did someone explain to you what this test result meant?
  - **(If No)** How did you feel about this?
  - Would you have preferred to know the result?
- If not, ask the following questions:

If the hospital had asked you to provide a poo sample, you are given a kit with a container for you to take home, collect a sample of your poo and send it back in a special envelope. The test looks for traces of blood in the poo, which can be a sign of bowel cancer (but not caused by cancer in all cases).

- - How would you have felt if your doctor had asked you to provide a poo sample, for them to do this test?
  - The hospital doctors might use the results of this test to decide how urgently you needed to have your investigations. Would you have been happy for the doctor to have used this test result to guide decisions about your care?
  - Would you still have been happy if it meant you had to wait longer to have a colonoscopy?

***FIT Testing***

Now we would like to talk to you about your thoughts on this type of test, more generally, if that is ok?

This test looks to see whether there are traces of blood in people’s poo, as this is a sign that people may have bowel cancer (although, most of those with blood in their poo won’t). This test measures the amount of blood in someone’s poo and produces a “score”. Patients with a low score may not be referred to hospital, but those with a higher score will.

- Do you think that this is a good way to help identify people who need to be seen in hospital and those who don’t?
- Do you think there are any benefits to using this test?
- What concerns do you have about it?
- Would you feel able to do the poo sample / test at home?
- Some people who would have previously been referred to hospital because of their symptoms, would now not be, because they have a low poo test score, and so are classed as being at ‘low’ risk of having cancer. How do you feel about this?
  - How do you think GPs should look after these patients?
- Are there circumstances when you think people who should go straight to hospital for a colonoscopy when they have bowel symptoms, without having to do a poo test?
- Do you think there are some people who would not want, or be able, to do the poo test? Who might they be? What could help them to do it?
- Why do you think some people might not complete the test with the kit?

**Interview Topic Guide: Health Care Practitioners**

This guide covers the general topics and questions that will covered in the interviews. It will be used flexibly, so that the participant can share information in a way that is most natural to them. Questions are intended to be used with appropriate probes as needed. How questions are asked and the order in which they will be asked will vary from interview to interview. It may also be the case that not all topics and questions are covered in every interview.  If a new topic of interest arises in one interview, this may be added to the guide for inclusion in subsequent interviews. Conversely, a topic or question that is found not to deliver useful information may be dropped from subsequent interviews.

***FIT Experience***

- To begin with, please can you tell us what your job role is, and the type of care you provide for / contact you have with patients with possible symptoms of colorectal cancer?
- What use do you make of FIT in your work with these patients?

Please can you describe for me what you understand a FIT does, or is for. What do the results mean? / What does this mean with respect to bowel cancer symptoms?

- Have you been involved in the delivery of FIT-based triage of patients with possible symptoms of colorectal cancer?

*If yes:*

- - What “groups” were the patients triaged into?
  - What was your role in this pathway?
- *If no:*
  - *Do you envisage being involved in this in future?*

***For practitioners who have delivered FIT triage***

- When did you start to use FIT to triage symptomatic patients?
- What do you think about the use of FIT as a means of triaging symptomatic patients?
  - What are the advantages?
  - Are there any disadvantages?
- Can you please explain, using a typical patient as an example, how you would use FIT in your assessment and decision-making process for symptomatic patients?
- Do you refer to any guidelines to guide your use of FIT-based triage in the symptomatic population?
  - What guidelines do they refer to?
  - How useful are they (or not) and why?
- Are there contexts in which you would deviate from the guidelines in your management of a patient?
- Are there any particular groups of patients whom you have concerns about in the context of FIT-based triage?
  - Who are they and why?
- Are there any instances, or patient groups, for whom you think it might not be appropriate to request sample for FIT?
  - Who are they and why?
  - Probe – what about patients in a younger age group / patients with suspected IBD / patients with rectal bleeding?
- Are there any instances in which you would prefer to obtain repeat FIT-scores to inform your decision-making in relation to that patient’s care?
  - Who are they and why?
  - How many repeat scores would you wish to obtain / over what time period?
- Has the introduction of FIT-based triage for symptomatic patients changed your clinical practice?
  - In what ways?
- Has the introduction of FIT-based triage for symptomatic patients changed your workload, or the workload of your wider team?
- Do you think FIT is – or will be - easily integrated into the appraisal and management pathways for symptomatic patients?
  - Is there anything about the integration of FIT into patient pathways which you think will be challenging (either in terms of your own practice or more generally)?
  - Is there anything that needs to be put in place so that this system of triage works well?
- Since you started to use FIT with symptomatic patients, have you encountered any problems in the delivery of this, from either the health care service, or patient, sides, more generally?
- Is there anything that could help you in relation to the use FIT in the symptomatic population, for instance, professional training, prompts during consultations, or guidelines?
- What do patients tell you about their experiences of using the FIT kit at home?
- Do patients ever not return complete their FIT test? What do you do (or would you do) in these instances?
- Do you think patients understand the purpose of FIT and its relevance to both their onward care, and possible cancer risk?
- Do you think patients find this use of FIT acceptable?
  - Probe – Do you ever experience negative reactions from patients?
    - What are these?
  - How do you communicate this to patients? Is there anything that could help you in this?
  - Do you think that triaging of symptomatic patients, using FIT testing, is beneficial:
  - For the health service
  - For patients
  - For patients with other underlying conditions?

***For practitioners who have NOT delivered FIT triage***

- Do you think that there are any advantages to FIT-based triage of symptomatic patients?
- Are there any disadvantages of FIT-based triage in the symptomatic population?
- Are you aware of any guidance for the use of FIT in the symptomatic population?
- Are there any instances in which you would imagine it would be appropriate to override these guidelines?
- Are there any instances in which you imagine it might not be appropriate, or possible, to get a patient to provide a sample for FIT?
  - Who are they and why?
  - Probe – what about patients in a younger age group / patients with suspected IBD / patients with rectal bleeding?
- Are there any instances in which you imagine it might be appropriate to ask a patient to repeat FIT to inform your decision-making in relation to that patient’s care?
- Do you think FIT is a useful tool to aid decision-making around patient care?
- Do you think FIT could be easily integrated into the appraisal and management pathways for symptomatic patients?
- Do you see any potential problems that could arise by introducing FIT for the management and appraisal of symptomatic patients?
- Do you see any potential challenges in the introduction of FIT, either with respect to your own practice or more generally?
- Is there anything that would help you in relation to the use FIT in the symptomatic population, for instance, professional training or prompts during consultations?
- Do you think that your patient population would be able, and willing, to complete FIT tests?
  - Do you anticipate there being reluctance to complete FIT from any particular patient groups?
- What would you do if a patient did not complete their FIT test?
  - What might be done to encourage patients to complete their tests?
- Do you think patients will understand the purpose of FIT and its relevance to both their onward care, and possible cancer risk?
  - How would you communicate this to patients? Is there anything that could help you in this?
- Do you think patients understand the purpose of FIT and its relevance to both their onward care, and possible cancer risk?
- Do you think patients find this acceptable?
  - How do you communicate this to patients? Is there anything that could help you in this?
  - Do you think that triaging of symptomatic patients, using FIT testing, is beneficial:
  - For the health service
  - For patients
