# Supplementary material for: Patient experiences and expectations of faecal immunochemical testing for investigation of colorectal cancer symptoms: a cross-sectional qualitative interview study with patients and practitioners in the UK
Source: BMJ Open. 2025 May 16;15(5):e093215. doi: 10.1136/bmjopen-2024-093215 (PMC12086939; doi:10.1136/bmjopen-2024-093215)
Supplement: online supplemental table 1 [file bmjopen-15-5-s002.docx]

**Supplementary Table 1: Additional Quotes**

| **Topic** | **Quote** | **Participant characteristics** |
| --- | --- | --- |
| Understanding the Test |  |  |
|  | *it was looking for blood in the stool. Which is, I know it’s a marker for cancer, I know it’s a marker for other things.* | *P001, male, early 70’s, FIT negative* |
|  | *it’s never the pleasantest of things to do, is it, but it wasn’t unduly difficult...It is just, how can you put it, a messy thing to do, not a pleasant thing…* | *P002, male, mid 70’s FIT positive* |
|  | *It actually looks for bowel cancer, I presume.* | *P009, female, mid 60’s, FIT negative* |
|  | *I think when you’re doing a FIT test and you explain why you’re doing it and what the implications are of a positive test I think cancer’s usually the thing that comes to the forefront of their thought, their minds and the thought process. So I think they’re probably quite easy to maybe jump to that is what we’re looking at specifically here rather than appreciating other causes of bleeding.* | *C013. GP* |
|  | *“I sometimes say, it’s a bit like the bowel screening test but it’s got a lower threshold to look for blood, so that’s what I say. So, I do say it’s a cancer screen because I think it makes it more likely for the patient to do it.”* | *C017, GP* |
|  | *I’m not so great at explaining it…in a 10-minute consultation you have to take a full history, … examine the patient and in the end quickly rush through, well this is what I want you to … so there’s not a lot of time to explain exactly what a FIT test is.* | *C018 GP* |
| Practicalities of Completion | *I think sometimes patients are reluctant to do a FIT test and I think sometimes patients, even though you’ve told them that it’s not the same as bowel cancer screening test, they still believe, well I’ve just done that recently, I’m not going to pick it up.* |  |
|  | *…the easiest thing that took place was that bowel test, the little stick, and if that’s all it takes to detect somebody having a problem, then it’s a miracle!* | *P010, female, mid 50’s, FIT negative* |
|  | *We do have people ringing us going, “I really don’t know what I’m doing”, and so we’ll talk them through the*  *process and try and help them as much as we can.* | *C011 Lab Director* |
|  | *even though you think your instructions are really simple, they’ll still send us more than we need and, yeah, we’ve had people where they’ve thought they’ve had to stick the probe up their bottom and things like that. Yeah, so it’s getting those instructions right.* |  |
|  | *“ …I vaguely remember it being about 78% returns….it would be good to understand the causation of this….it* [the non-completion rate] *was about 20%”* |  |
|  | *There are spoilt returns. We get some real weird and wonderful samples, believe me! What we get in a bag sometimes you would not believe.* | *C012 Consultant Biochemist* |
|  | *Nobody’s that keen on it but I think they understand the value of it. It’s something that, it’s certainly easier to do than the old-style FOB tests because they only have to do it once. Generally, I would say it appears to be accepted by patients as part of their diagnostic pathway, I think they’re generally pretty happy to do it if it’s advised.* | *C016, Consultant Nurse* |
|  | *There’s the visually impaired, I think they really struggle to do the test.”* | *P028, Pathology Lab Director* |
| Understanding the Result | *That came back clear that there was no blood in my stool.* | *P001, male, early 70’s, FIT negative* |
|  | *Still uncertain, yeah, …I wasn’t hundred percent sure there was nothing there* | *P015, female, late 70’s, FIT positive* |
|  | *…I’d always sort of caveat me giving it to them with a “if this is normal your chance of having a cancer is like one in ten thousand at least so it essentially rules it out” …* | *C013, GP* |
|  | *Once they know they’ve been referred on a two week wait suspected bowel cancer pathway* [a pathway which means the patient will be seen urgently in secondary care] *they think that they’ve got a positive test for bowel cancer, in some cases, obviously not all. Some patients become very anxious that that’s the case.* | *C016, Consultant Nurse* |
|  | *whenever you refer anyone on a two-week wait (pathway)* [a pathway which means the patient will be seen urgently in secondary care]*, I think you do automatically cause some degree of panic. … When you’ve been told that … I need to refer you on a two week wait because potentially this could be cancer. I think they hear that and even when you turn round, …and say well actually through I send quite a lot of people with FIT positive and they don’t turn out to have cancer, I don’t think people hear that because they heard cancer and they stop. …it’s only natural that you’re going to panic when the word cancer has been mentioned and you’re being checked.* | *C020, GP* |
| Expectations for Referral and Investigation | *colonoscopy examines part of your body. … it must be more accurate. I would think. It’s actually seeing. … it’s examining at close quarters .... So, if that doesn’t spot anything, what would?* | *P001, male, early 70’s, FIT negative* |
|  | *I was hoping for an investigation to see whether it was cancer. I was expecting the referral at that point…. That’s what I was hoping for, yes.* | *P007, female, late 60’s, FIT positive* |
|  | *the FIT test, it’s only like a stool sample and sometimes I daresay they might miss things...* | *P009, female, mid 60’s, FIT negative* |
|  | *I wanted the referral because I was really concerned that it might be bowel cancer and I was actually frightened I’d left it too late.* | *P012, female, mid 40’s, FIT positive* |
|  | *they said the other alternative would be for a colonoscopy to make sure and I said, well, I’d rather have a colonoscopy, just to make sure.* | *P015, female, late 70’s, FIT positive* |
|  | *I think perhaps even if it hadn’t showed any blood, they might have still sent me for a colonoscopy because of me and my history… but, it’s obviously somebody’s judgement and that wasn’t obviously mine…I mean the people who’s making the judgement have more knowledge than I have haven’t they so basically it’s not something I could really dispute.* | *P017, male, mid 70’s, FIT positive* |
|  | *I thought ah well, if that comes back negative, which I was expecting it to come back negative, where do I go from there? Because … to me if that had come back negative, she wasn’t going to send us down the path that I went [for colonoscopy].* | *P019, female mid 60’s, FIT positive* |
|  | *you just want, you want the doctor to go, oh, I know what’s caused it, it’s this, it’s nothing serious, you know, whatever, it’ll go away in a couple of days or whatever. I suppose I wanted him to, yeah, do some tests. I didn’t know what the tests would be. I was expecting a blood test. I didn’t think they would refer me to get a colonoscopy.* | *P020, male, late 30’s, FIT negative* |
|  | *especially with rectal bleeding they wanted to know what was causing it. They (the patients) wanted to know the cause of the rectal bleeding. They actively wanted a consultation and an investigation other than FIT when that was the particular symptom.* | *C001, Surgeon* |
|  | *there is a slight fear I think from patients that they do a stool test rather than a test that’s looked in their colon. But I think that a lot of that fear is borne out of the fact that again, a lot of the literature and a lot of the publications that patients see, etc, will talk about missing the odd cancer at the low FIT level. But does anybody ever tell them that colonoscopy will miss a cancer as well? Probably not, we should be.* | *C003, Radiologist* |
|  | *…not a lot of people like having colonoscopies so if they can avoid that and still get a pretty good outcome out of it then that’s ideal.* | *C013, GP* |
