# Supplementary material for: Patient experiences and expectations of faecal immunochemical testing for investigation of colorectal cancer symptoms: a cross-sectional qualitative interview study with patients and practitioners in the UK
Source: BMJ Open. 2025 May 16;15(5):e093215. doi: 10.1136/bmjopen-2024-093215 (PMC12086939; doi:10.1136/bmjopen-2024-093215)
Supplement: Supplementary file 3 [file bmjopen-2024-093215.reviewer_comments.pdf]

PEER REVIEW HISTORY

BMJ Open publishes all reviews undertaken for accepted manuscripts. Reviewers are asked to complete a checklist review form and are provided with free text boxes to elaborate on their assessment. These free text comments are reproduced below.

ARTICLE DETAILS

Title (Provisional)

Patient experiences and expectations of faecal immunochemical testing for investigation of colorectal cancer symptoms: a cross-sectional qualitative interview study with patients and practitioners in the UK.

Authors

Biran, Adam; Dobson, Christina; Rees, Colin J.; Hamilton, Willie; Humes, David; Neilson, Laura Jane; Turvill, James; von Wagner, Christian; Whelpton, John; Sharp, Linda

---

VERSION 1 - REVIEW

Reviewer 1

Name Harris, Dean

Affiliation Abertawe Bro Morgannwg University Health Board, Department of Colorectal Surgery

Date 16-Oct-2024

COI Co-founder of a colorectal liquid biopsy test company

Abstract results misleading as written: “FIT completion was unproblematic”, then next sentence “HPs expressed concerns over non-completion rates”. So completion is a problem. Amend first sentence to “conduct of FIT was unproblematic”? Needs context from whose perspective it is unproblematic from.

Is there a risk that the health professionals being recruited from a guideline development group were a biased population, given that the guideline supported the use of FIT?

Methods

Study information sheet: translated?

Was there purposive sampling to seek over-representation of underserved groups who have increased barriers to FIT completion? Eligibility criteria was the successful completion of FIT, so the study excludes those who were non-compliant with FIT (hence “FIT completion was unproblematic”?)

One male conducted the patient interviews: risk of bias should be mentioned as no matching of interviewer to patient based on protected characteristics to enable information sharing? Interviewers for the HCPs are detailed only.

Were guidelines such as INCLUDE and PIRIT checklists followed for the PPI?

Was the data saturation approach applied to both patients and HCPs or just HCPs? Line 48 page 4

Ethics: Health Care Wales should be Health and Care Research Wales (HCRW)

Description of the PPIE is lacking. Did the PPIE review the interview schedule/questions and the transcripts?

No table of patient demographics, especially ethnicity, given that this was the reason for the geographic areas chosen.

What number/proportion of "participating patients found the test easy to complete"? page 6 line 53

Discussion

The statement "We found no evidence for substantial barriers to the use of FIT-based referral" needs to be qualified with the fact that only patients who successfully completed a FIT were included.

A section on limitations of the study should be included.

No inclusion of FIT non-compliance literature in discussion/references, nor other papers which investigated FIT among symptomatic patients eg https://doi.org/10.1111/hex.13796

Reviewer 2
Name Mowat, Craig
Affiliation Division of Population Health and Genomics, Ninewells Hospital and Medical School, University of Dundee
Date 24-Oct-2024
COI None

This is interesting but not novel.

It transpires this paper is one half of a retrospective study looking at experiences and expectations of patients and healthcare workers in the feasibility and acceptability of FIT-based triage of bowel symptoms in primary care. This details the patient experience whereas the other half of the data has been salami sliced into another unpublished paper (reference 12) which is not accessible to the reader/reviewer which is frustrating. Why not report this as one paper?

My other main constructive comment is that there is no paragraph in the Discussion to address strengths and limitations of the paper. References 9 and 10 have already studied this area and with much larger numbers, and were conducted prospectively, but not discussed or compared. In the current study patients were approached some months after the event. Worthy of discussion.

Minor comments:

The abstract doesn't clearly explain the study aims

There is a typo in the Abstract Methods section

You write about anecdotal concerns of FIT not return estimated at '20%' - with no evidence to corroborate - and reiterate these concerns in the Discussion. Is there a way to measure FIT non-return rate?

VERSION 1 - AUTHOR RESPONSE

Reviewer 1 comments	Action taken
Abstract results misleading as written: “FIT completion was unproblematic”, then next sentence “HPs expressed concerns over non-completion rates”. So completion is a problem. Amend first sentence to “conduct of FIT was unproblematic”? Needs context from whose perspective it is unproblematic from	The abstract has been amended and now reads as follows:<br><br>*Completion of FIT was unproblematic from the perspective of patients who returned the test. However, health professionals expressed concern over non-return rates.*
Is there a risk that the health professionals being recruited from a guideline development group were a biased population, given that the guideline supported the use of FIT?	We believed that guidelines group members would be particularly informative participants, having considered FIT more deeply, and often having had more extensive experience of symptomatic FIT, than the wider population of health professionals. It is possible that the views of guideline group members are not representative of the wider population. We do not consider this problematic in the context of the current results which pertain primarily to patient experiences. Nevertheless, we agree that the reader should be aware of this characteristic of the sample. We therefore now refer to this specifically in our study limitations as follows:

	• *Our sample of health professionals included members of the BSG/ACPGBI Symptomatic FIT Guideline Development Group whose views and experiences may not be representative of a wider population of health professionals.*<br><br>We have also added the following sentence to the Results section to make clear the extent of the sample comprising group members:<br><br>*Ten of these participants had been members of the BSG/ACPCBI Symptomatic FIT Guideline Development Group.*
Study information sheet: translated?	This small study lacked sufficient funding for interpreters and translation. It was therefore and eligibility requirement for participants to sufficient English language proficiency.<br><br>We have amended the Methods section as follows to make this clear:<br><br>*Participants were required to be sufficiently proficient in English language to take part in an interview, without an interpreter.*<br><br>*Eligible patients were provided with a study information sheet and consent form (both documents in English).*
Was there purposive sampling to seek over-representation of underserved groups who have increased barriers to FIT completion? Eligibility criteria was the successful completion of FIT, so the study excludes those who were non-compliant with FIT (hence "FIT completion was unproblematic"?)	There was no purposive sampling of underserved groups as our recruitment process did not yield sufficient potential participants from these groups. We acknowledge the lack of ethnic diversity in our statement of study limitations.<br><br>We agree that the experiences of those who did not complete FIT will be different from those who did and who were included as participants. We acknowledge this in our statement of study limitations.

One male conducted the patient interviews: risk of bias should be mentioned as no matching of interviewer to patient based on protected characteristics to enable information sharing? Interviewers for the HCPs are detailed only.	As AB interviewed HCPs and patients, his details are provided. We have amended the Methods section as follows so as to ensure that the reader is made aware of the possibility that interviewer gender may affect data:<br><br>*Patient interviews were conducted by AB only, accepting the possibility that this might have a differential effect on the data provided by male and female respondents.*
Were guidelines such as INCLUDE and PIRIT checklists followed for the PPI?	Patient and public involvement has been a core thread of this research study (and the entire research programme), taking an inclusive and holistic approach to the inclusion of people with lived experience in the oversight and delivery of this work. We have followed best practice principles as set out in the UK standards for public involvement, involving panel members in meaningful ways and ensuring fair recompense, in line with INVOLVE rates of payment for public representatives.<br><br>We have now added the following text to the manuscript to outline our approach to PPI to the reader:<br><br>*A patient panel of individuals with experience of FIT and endoscopy has contributed to study oversight from conception to dissemination, with best practice principles, as set out in the UK Standards for Public Involvement, followed throughout. Specifically, panel members reviewed patient-facing study documents (information sheet, consent form), and draft interview guides, contributed to interpretation of data and theme development, and commented on an earlier draft of the manuscript.*
Was the data saturation approach applied to both patients and HCPs or just HCPs? Line 48 page 4	We have amended the Methods section as follows to make clear that the same approach was applied to both participant groups:<br><br>*Data collection ceased when the researchers (AB and CD) believed that additional interviews*

	with either participant group were no longer generating data that would contribute to additional insights.
Ethics: Health Care Wales should be Health and Care Research Wales (HCRW)	This has been corrected as follows:<br><br>*The study received a favourable opinion from HRA and Health and Care Research Wales (HCRW) on 11th August 2022 (REC reference 22/NW/0240).*
Description of the PPIE is lacking.<br><br>Did the PPIE review the interview schedule/questions and the transcripts?	The Methods section previously stated that draft data collection tools were reviewed. We have amended this as follows to make it clear that this refers to review of interview questions and that review did not extend to transcripts:<br><br>*Specifically, panel members reviewed patient-facing study documents (information sheet, consent form), and draft interview guides, contributed to interpretation of data and theme development, and commented on an earlier draft of the manuscript.*
No table of patient demographics, especially ethnicity, given that this was the reason for the geographic areas chosen.	Lack of ethnic diversity is acknowledged in the study limitations. Patient demographics are now outlined in the text, with specific reference to ethnicity, as follows:<br><br>*We interviewed 21 patients (12 women, 9 men) who had experienced symptoms of possible CRC, completed a FIT in primary care and undergone colonoscopy. They had a median age of 67 years (range 28-86); 20 identified as White.*
What number/proportion of "participating patients found the test easy to complete"? page 6 line 53	In this small, qualitative study, -we believe it would be potentially misleading to present results and numbers or proportions. However, reflecting on the reviewer's comment, we have amended the sentence so that it refers to patients not having significant difficulties, thereby providing a more precise representation of the data.:<br><br>*Consistent with previous studies of FIT in symptomatic populations [9,10], and asymptomatic screening populations [7,8],*

	none of our participants reported significant difficulties with completing FIT sampling.
Discussion<br>The statement "We found no evidence for substantial barriers to the use of FIT-based referral" needs to be qualified with the fact that only patients who successfully completed a FIT were included.	We have added a qualifier to the statement as follows:<br><br>*We found no evidence for substantial barriers to the use of FIT-based referral, either in relation to test completion or acceptability of it as a triage tool, within this participant group who had completed FIT.*
A section on limitations of the study should be included.	This section has been included immediately following the abstract as per the journal requirements.
No inclusion of FIT non-compliance literature in discussion/references, nor other papers which investigated FIT among symptomatic patients eg https://doi.org/10.1111/hex.13796	We thank the reviewer for drawing our attention to this study which we have now cited.<br><br>As ours was not a study of non-compliance we have not included discussion of this literature;, however, we do draw on limited references from studies of FIT in screening.
**Reviewer 2 comments**	**Action taken**
that there is no paragraph in the Discussion to address strengths and limitations of the paper.	This section has been included immediately following the abstract as per the journal requirements.
References 9 and 10 have already studied this area and with much larger numbers, and were conducted prospectively, but not discussed or compared. In the current study patients were approached some months after the event. Worthy of discussion.	We draw on comparisons with both references in our discussion, although it should be noted that study 10, while larger, uses a different methodology.
There is a typo in the Abstract Methods section	We are unsure what typo the reviewer is referring to. We have slightly revised the Abstract to address reviewer comments and have checked it carefully for typos.
The abstract doesn't clearly explain the study aims	We have amended a sentence in the Abstract to make it clear that this refers to the study aims as follows:

	In this qualitative study we aimed to understand the feasibility and acceptability of FIT-based triage for patients.
You write about anecdotal concerns of FIT not return estimated at '20%' - with no evidence to corroborate - and reiterate these concerns in the Discussion. Is there a way to measure FIT non-return rate?	Measuring FIT non-return rate should be possible and useful, but is beyond the scope of this study.<br><br>We have amended the reporting of results as follows to make clear the source of the estimate:<br><br>*Nevertheless, non-completion, or non-return of samples was a concern for health professionals, with one laboratory director estimating a non-return rate of around 20%.*

VERSION 2 - REVIEW

Reviewer 1

Name Harris, Dean

Affiliation Abertawe Bro Morgannwg University Health Board, Department of Colorectal Surgery

Date 20-Mar-2025

COI

All previous issues corrected in R1.
